# Complete plastome sequence of *Dracaena cambodiana* (Asparagaceae): a species considered “Vulnerable” in Southeast Asia

**DOI:** 10.1080/23802359.2018.1473740

**Published:** 2018-05-21

**Authors:** Zhi-Xin Zhu, Wei-Xue Mu, Jian-Hua Wang, Jin-Ran Zhang, Kun-Kun Zhao, Cynthia Ross Friedman, Hua-Feng Wang

**Affiliations:** a Hainan Key Laboratory for Sustainable Utilization of Tropical Bioresources, Institute of Tropical Agriculture and Forestry, Hainan University, Haikou, China;; b BGI-Shenzhen, Shenzhen, China;; c Department of Botany, University of British Columbia, Vancouver, Canada

**Keywords:** *Dracaena cambodiana*, illumina sequencing, plastome, Asparagaceae, phylogenetic analysis, Asparagales

## Abstract

*Dracaena cambodiana* (Asparagaceae) is a treelike plant ranging from 3 to 10 m tall. It grows in low-elevation forests (0–300 m) having dry and sandy soils. It is distributed in Southern Hainan Island in China and other Southeast Asian countries (e.g. Cambodia, Laos, Thailand and Vietnam). The dried resin can be used medicinally as a substitute for that of *Dracaena cochinchinensis*. It has been ranked as a Vulnerable (VU) species in China. Here we report and characterize the complete plastid genome sequence of *D. cambodiana*. The complete plastome is 156,697 bp in length. It contains the typical structure and gene content of angiosperm plastomes, including two Inverted Repeat (IR) regions of 26,526 bp, a Large Single-Copy (LSC) region of 84,988 bp and a Small Single-Copy (SSC) region of 18,657 bp. The plastome contains 113 genes, consisting of 76 unique protein-coding genes, 30 unique tRNA genes, four unique rRNA genes and three pseudogenes (i.e. *matK, infA, ndhF*). The overall A/T content in the plastome of *D. cambodiana* is 62.4%. We performed phylogenetic analyses using the entire plastome, including spacers, introns, etc., and we determined that *D. cambodiana* and *Maianthemum bicolor* were closely related. The complete plastome sequence of *D. cambodiana* will provide a useful resource for the conservation genetics of this species as well as for phylogenetic studies in Asparagales.

*Dracaena cambodiana* Pierre ex Gagnep. (Asparagaceae) is a treelike plant ranging from 3 to 10 m tall. It grows in low-elevation forests (0–300 m) having dry and sandy soils. It is distributed in Southern Hainan Island in China and other southeast Asian countries (e.g. Cambodia, Laos, Thailand, and Vietnam). The dried resin can be used medicinally as a substitute for that of *D. cochinchinensis* (Chen et al. 2000). *Dracaena cambodiana* has been ranked as a Vulnerable (VU) species in China (Ministry of Environmental Protection of the Peoples Republic of China 2013). Consequently, genetic and genomic information is urgently needed to aid its conservation. Here, we report and characterize the complete plastome of *D. cambodiana* (GenBank accession number: MH293451, this study) based on Illumina paired-end sequencing data. This is the first report of a complete plastome for the genus *Dracaena*.

In this study, *D. cambodiana* was sampled from the campus of Hainan University in Hainan province of China (110.33°E, 20.06°N). A voucher specimen (Wang et al. B248) was deposited in the herbarium of the Institute of Tropical Agriculture and Forestry (code of herbarium: HUTB), Hainan University, Haikou, China.

The modified cetyltrimethylammonium bromide (CTAB) protocol of Doyle and Doyle (1987) was used to extract genomic DNA from dry leave tissues. The genomic DNA of each sample was quantified and analyzed with Agilent 200 BioAnalyzer. Samples yield at least 0.8 μg DNA were selected for subsequent libraries construction and de novo sequencing. Genomic DNA of selected samples were used to build the paired-end libraries with 200-400bp insert size. Libraries were sequenced using BGISEQ-500 platform at BGI Shenzhen, China and produced about 8 Gb high quality per sample with 100 bp paired-end reads. Raw reads were trimmed using SOAPfilter_v2.2 with the following criteria (1) reads with >10 percent base of N; (2) reads with >40 percent of low quality (value <= 10); (3) reads contaminated by adaptor and produced by PCR duplication. Around 6 Gb clean data for each sample were used to perform the assembling of chloroplast genome using MITObim v1.8 (Hahn et al. 2013). Cleaned reads were assembled against the plastome of Nolina atopocarpa (KX931462) (McKain et al. 2016) using MITO bim v1.8 (Hahn et al. 2013). Plastomes were annotated using Geneious R8.0.2 (Biomatters Ltd., Auckland, New Zealand) against the plastome of *Nolina atopocarpa* (Genbank Accession number: KX931462). DOGMA (Wyman et al. 2004) was used to correct the annotation, and OGDRAW (http://ogdraw.mpimp-golm.mpg.de/) (Lohse et al. 2013) was used to generate the circular plastome map.

The plastome of *D. cambodiana* possessed a total length 156,697 bp with the typical quadripartite structure of angiosperms, containing two Inverted Repeats (IRs) of 26,526 bp separated by a large single-copy (LSC) region and a small single-copy (SSC) region of 84,988 and 18,657 bp, respectively. The plastome was found to contain 113 genes, including 76 protein-coding genes (seven of which are duplicated in the IR), 4 ribosomal RNA genes, 30 tRNA genes (eight of which are duplicated in the IR), and 3 pseudogenes (*matK, infA, ndhF*). Among these genes, 14 genes (*trnA-UGC, trnG-GCC, trnI-GAU, trnK-UUU, trnL-UAA, trnV-UAC*, *atpF, ndhA, ndhB, petB, petD, rpoC1, rpl2, rpl16, rps16*) harboured a single intron, and three genes (*ycf3, clp*P*, rps12*) had two introns. The gene *rps12* has trans-splicing. The overall A/T content of the plastome was 62.4%, while those of the LSC, SSC, and IR regions were 64.3%, 68.9%, and 57%, respectively.

We used RAxML (Stamatakis 2006) with 1000 bootstraps under the GTRGAMMAI substitution model to reconstruct a maximum likelihood (ML) phylogeny of 12 published complete plastomes of Asparagaceae, using *Agapanthus coddii* (Amaryllidaceae, Asparagales) as an outgroup. The phylogenetic analysis indicated that *D. cambodiana* and *Maianthemum bicolor* are closely related (Figure 1). Furthermore, all members of Asparagaceae were clustered with a high bootstrap support (BS) value. With the plastome of *D. cambodiana* plastome now at hand, its conservation value can be better assessed, and phylogenetic studies of Asparagales can be explored more fulsomely.

**Figure 1.**
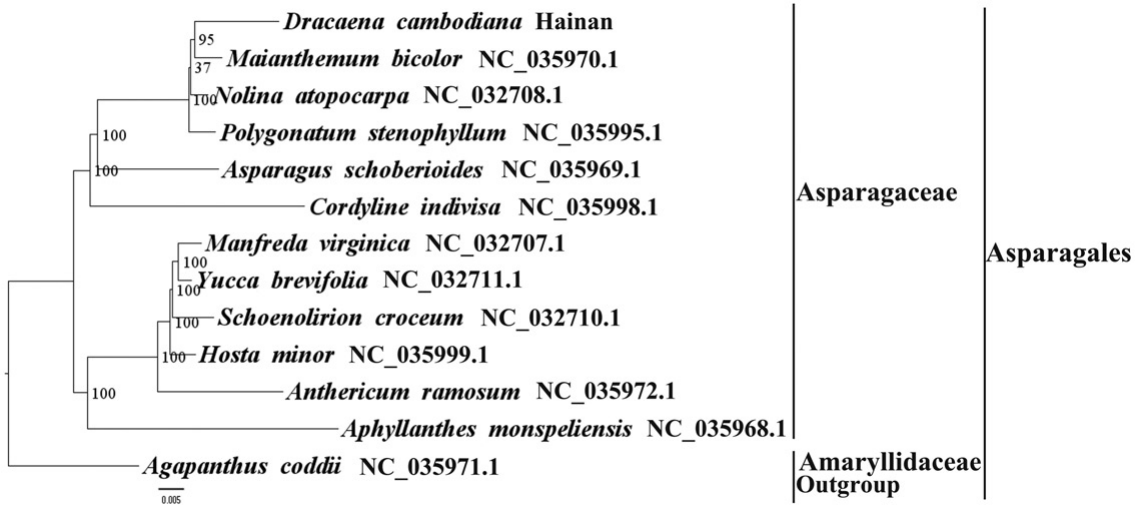
The best ML phylogeny recovered from 13 complete plastome sequences by RAxML. Accession numbers: *D. cambodiana* (GenBank accession number: MH293451, this study), *Maianthemum bicolor* NC_035970.1, *Nolina atopocarpa* NC_032708.1*, Polygonatum stenophyllum* NC_035995.1, *Asparagus schoberioides* NC_035969.1, *Cordyline indivisa* NC_035998.1, *Manfreda virginica* NC_032707.1*, Yucca brevifolia* NC_032711.1, *Schoenolirion croceum* NC_032710.1, *Hosta minor* NC_035999.1, *Anthericum ramosum* NC_035972.1, *Aphyllanthes monspeliensis* NC_035968.1, *Agapanthus coddii* NC_035971.1.
